# Polygenic Risk Score, Environmental Tobacco Smoke, and Risk of Lung Adenocarcinoma in Never-Smoking Women in Taiwan

**DOI:** 10.1001/jamanetworkopen.2023.39254

**Published:** 2023-11-13

**Authors:** Batel Blechter, Li-Hsin Chien, Tzu-Yu Chen, I-Shou Chang, Parichoy Pal Choudhury, Chin-Fu Hsiao, Xiao-Ou Shu, Jason Y. Y. Wong, Kuan-Yu Chen, Gee-Chen Chang, Ying-Huang Tsai, Wu-Chou Su, Ming-Shyan Huang, Yuh-Min Chen, Chih-Yi Chen, Hsiao-Han Hung, Jia-Wei Hu, Jianxin Shi, Wei Zheng, Anne F. Rositch, Chien-Jen Chen, Nilanjan Chatterjee, Pan-Chyr Yang, Nathaniel Rothman, Chao Agnes Hsiung, Qing Lan

**Affiliations:** 1 Division of Cancer Epidemiology and Genetics, National Cancer Institute, National Institutes of Health, Rockville, Maryland; 2 Institute of Population Health Sciences, National Health Research Institutes, Zhunan, Taiwan; 3 Department of Applied Mathematics, Chung Yuan Christian University, Zhongli, Taiwan; 4 National Institute of Cancer Research, National Health Research Institutes, Zhunan, Taiwan; 5 Now with American Cancer Society, Kennesaw, Georgia; 6 Division of Epidemiology, Department of Medicine, Vanderbilt University Medical Center, Nashville, Tennessee; 7 Now with Epidemiology and Community Health Branch, National Heart Lung and Blood Institute, Bethesda, Maryland; 8 Department of Internal Medicine, National Taiwan University Hospital and College of Medicine, National Taiwan University, Taipei, Taiwan; 9 School of Medicine and Institute of Medicine, Chung Shan Medical University, Taichung, Taiwan; 10 Division of Pulmonary Medicine, Department of Internal Medicine, Chung Shan Medical University Hospital, Taichung, Taiwan; 11 Institute of Biomedical Sciences, National Chung Hsing University, Taichung, Taiwan; 12 Division of Chest Medicine, Department of Internal Medicine, Taichung Veterans General Hospital, Taichung, Taiwan; 13 Department of Respiratory Therapy, Chang Gung University, Taoyuan, Taiwan; 14 Department of Pulmonary and Critical Care, Xiamen Chang Gung Hospital, Xiamen, China; 15 Department of Oncology, National Cheng Kung University Hospital, College of Medicine, National Cheng Kung University, Tainan, Taiwan; 16 Department of Internal Medicine, E-Da Cancer Hospital, School of Medicine, I-Shou University, Kaohsiung, Taiwan; 17 School of Medicine, National Yang Ming Chiao Tung University, Taipei, Taiwan; 18 Department of Chest Medicine, Taipei Veterans General Hospital, Taipei, Taiwan; 19 Institute of Medicine, Chung Shan Medical University Hospital, Taichung, Taiwan; 20 Division of Thoracic Surgery, Department of Surgery, Chung Shan Medical University Hospital, Taichung, Taiwan; 21 Department of Epidemiology, Johns Hopkins Bloomberg School of Public Health, Baltimore, Maryland; 22 Genomics Research Center, Academia Sinica, Taipei, Taiwan; 23 Department of Biostatistics, Johns Hopkins Bloomberg School of Public Health, Baltimore, Maryland

## Abstract

**Importance:**

Estimating absolute risk of lung cancer for never-smoking individuals is important to inform lung cancer screening programs.

**Objectives:**

To integrate data on environmental tobacco smoke (ETS), a known lung cancer risk factor, with a polygenic risk score (PRS) that captures overall genetic susceptibility, to estimate the absolute risk of lung adenocarcinoma (LUAD) among never-smokers in Taiwan.

**Design, Setting, and Participants:**

The analyses were conducted in never-smoking women in the Taiwan Genetic Epidemiology Study of Lung Adenocarcinoma, a case-control study. Participants were recruited between September 17, 2002, and March 30, 2011. Data analysis was performed from January 17 to July 15, 2022.

**Exposures:**

A PRS was derived using 25 genetic variants that achieved genome-wide significance (*P* < 5 × 10^−8^) in a recent genome-wide association study, and ETS was defined as never exposed, exposed at home or at work, and exposed at home and at work.

**Main Outcomes and Measures:**

The Individualized Coherent Absolute Risk Estimator software was used to estimate the lifetime absolute risk of LUAD in never-smoking women aged 40 years over a projected 40-year span among the controls by using the relative risk estimates for the PRS and ETS exposures, as well as age-specific lung cancer incidence rates for never-smokers in Taiwan. Likelihood ratio tests were conducted to assess an additive interaction between the PRS and ETS exposure.

**Results:**

Data were obtained on 1024 women with LUAD (mean [SD] age, 59.6 [11.4] years, 47.9% ever exposed to ETS at home, and 19.5% ever exposed to ETS at work) and 1024 controls (mean [SD] age, 58.9 [11.0] years, 37.0% ever exposed to ETS at home, and 14.3% ever exposed to ETS at work). The overall average lifetime 40-year absolute risk of LUAD estimated using PRS alone was 2.5% (range, 0.6%-10.3%) among women never exposed to ETS. When integrating both ETS and PRS data, the estimated absolute risk was 3.7% (range, 0.6%-14.5%) for women exposed to ETS at home or work and 5.3% (range, 1.2%-12.1%) for women exposed to ETS at home and work. A super-additive interaction between ETS and the PRS (*P* = 6.5 × 10^−4^ for interaction) was identified.

**Conclusions and Relevance:**

This study found differences in absolute risk of LUAD attributed to genetic susceptibility according to levels of ETS exposure in never-smoking women. Future studies are warranted to integrate these findings in expanded risk models for LUAD.

## Key Points

**Question:**

Can data on environmental tobacco smoke (ETS) and polygenic risk score (PRS) be used to estimate the absolute risk of lung adenocarcinoma (LUAD) among never-smoking women in Taiwan?

**Findings:**

In this case-control study of 1024 never-smoking women with LUAD and 1024 controls in Taiwan, a significant super-additive interaction between PRS and ETS exposure was observed. The estimated absolute risk of LUAD was higher among those with ETS exposure and high genetic susceptibility compared with those with ETS exposure and low genetic susceptibility.

**Meaning:**

This study presents preliminary findings for the stratification of absolute risk of LUAD in never-smoking women in Asia, which may be used in future risk modeling.

## Teaser

This case-control study estimates the risk of lung adenocarcinoma among never-smoking women in Taiwan using data on environmental tobacco smoke and a polygenic risk score.

## Introduction

Lung cancer is a major global health problem since it is the leading cause of cancer death worldwide, representing about 20% of all cancer mortality in 2020.^1^ While smoking is the most common cause of lung cancer, nearly 25% of patients with lung cancer are never-smokers, with the proportion varying geographically.^2^

In East Asia, where individuals are exposed to a variety of carcinogenic compounds from environmental, occupational, and domestic sources, the incidence rate of lung cancer among never-smokers is especially high. In particular, environmental tobacco smoke (ETS) is one of the leading risk factors for lung cancer among never-smokers.^3,4^ Environmental tobacco smoke contains genotoxic and carcinogenic byproducts of cigarette smoke, which are the main cause of lung adenocarcinoma (LUAD),^5,6^ the most common histologic subtype among never-smokers.^7,8^ In addition to environmental carcinogens, several authors of the present study recently conducted the largest multi-ancestry genome-wide association study (GWAS) of lung cancer among East Asian and European populations and identified 25 single nucleotide variants (SNVs) associated with LUAD among East Asian individuals, which highlights the importance of genetic susceptibility to the etiology of LUAD.^9^

The US Preventive Services Task Force recommends screening with low-dose computed tomography scan in current and former heavy smokers.^10^ Comparable lung cancer screening guidelines for heavy smokers have been adopted in mainland China, South Korea, Japan, and Taiwan.^11,12^ These guidelines were largely informed by the National Lung Screening Trial,^13^ which investigated high-risk current and former smokers, and the Dutch-Belgian Randomized Lung Cancer Screening Trial (NELSON).^14^ However, few lung cancer screening recommendations exist for never-smokers even though they make up a considerable proportion of newly diagnosed LUAD cases.^2,15^

Accurate estimation of absolute risk of lung cancer among never-smokers is important to inform future lung cancer screening programs for this susceptible population. While the associations between ETS, genetic susceptibility, and relative risk of lung cancer have been investigated, there has been limited work on the integration of these risk factors to better estimate the absolute risk of lung cancer in never-smokers. Developing absolute risk models by integrating genetic and environmental exposures can help identify individuals who are at higher risk for lung cancer to target primary and secondary disease prevention strategies, such as prioritizing reduction of environmental exposures and screening.^16^ Furthermore, we are able to assess how the absolute risk due to ETS exposure varies by level of genetic susceptibility. We conducted a preliminary study of never-smoking Asian women from the Genetic Epidemiological Study of Lung Adenocarcinoma (GELAC) to estimate the absolute risk of LUAD by integrating data on ETS exposure and genetic susceptibility, as well as test for gene-environment interactions.

## Methods

### Study Population

This study used the case-control component of GELAC in Taiwan, which aimed to evaluate the associations between questionnaire-based and genetic risk factors and lung cancer. The study design, questionnaire, and case ascertainment procedures of GELAC have been previously described in detail.^17^ Briefly, patients with lung cancer included ethnically Han Chinese individuals who were aged 18 years or older with histologically confirmed incident primary lung cancer. Patients were recruited between September 17, 2002, and March 30, 2011, from 6 medical centers. Individuals categorized as controls were matched on age (±2 years), gender, and ethnic background and were cancer-free and randomly selected from the health-examination departments during the same time and from the same 6 hospitals as case recruitment. We restricted our study to female lifetime never-smokers, defined as a woman who had never smoked or smoked less than 1 cigarette per week for 6 months at any period during her lifetime, and analysis included only those with LUAD. All participants provided written informed consent, and the study received approval from the institutional review boards of each hospital and the National Health Research Institutes in Taiwan. Participants did not receive financial compensation. This study followed the Strengthening the Reporting of Observational Studies in Epidemiology (STROBE) reporting guideline.

### Environmental and Genetic Exposures

A structured questionnaire was administered at recruitment, in which information on demographic characteristics and exposure to ETS at home and at work was collected.^17^ Environmental tobacco smoke exposure at home and at work were defined as regular exposure to tobacco smoke by a spouse and regular exposure while working with a smoker. Separate dichotomous variables for exposure to ETS at home and exposure to ETS at work were defined as never vs ever exposed.

Details on genotyping and quality control were previously described in detail.^18,19^ In brief, the samples in GELAC were partly genotyped using Illumina 660W^18^ at the US National Cancer Institute’s Cancer Genomics Research Laboratory.^18^ Genotype imputation was conducted using IMPUTE2 software, version 2.2.2, and the 1000 Genomes Project Phase 1, version 3, data were used as the reference panel.^20^ The GWAS data are deposited at dbGAP.^21^

### Statistical Analysis

Data analysis was conducted from January 17 to July 15, 2022. We derived a polygenic risk score (PRS) using 25 independent SNVs that have previously reached genome-wide significance (ie, *P* = 5 × 10^−8^) in a recently conducted 2-stage GWAS with 21 658 cases of histologically confirmed LUAD in East Asian populations from Taiwan, Mainland China, Japan, Singapore, and Korea.^9^ The PRS was calculated for each individual (i) as the following linear combination:
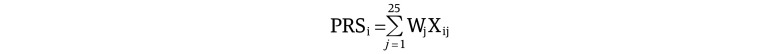
where X_ij_ is the number of risk alleles (ie, 0, 1, 2) in the *j*-th SNV for the *i*-th patients, and W_j_ is the weight for the *j*-th SNV.^22^ The weight for each SNV was the log-odds ratio of its association with LUAD risk from the previous GWAS. The list of the 25 SNVs and corresponding weights used to calculate the PRS are provided in eTable 1 in Supplement 1. We used logistic regression to estimate the association between exposure to ETS at home (never vs ever), exposure to ETS at work (never vs ever), PRS, and LUAD, adjusting for age (continuous) and the first 10 principal component scores to control for population stratification.

We used the Individualized Coherent Absolute Risk Estimator (iCARE) software^23,24^ to estimate the lifetime absolute risk of LUAD in never-smoking women aged 40 years over a projected 40-year span among the controls (n = 1024) by using the relative risk estimates for the PRS and ETS exposures, as well as age-specific lung cancer incidence rates for never-smokers in Taiwan (eTable 2 in Supplement 1).^25^ The iCARE software fits a model for absolute risk following the Cox proportional hazards model, assuming the age-specific incidence rates of the disease given a set of risk factors.^23,24^ Cumulative 40-year absolute risk of LUAD was calculated overall and by deciles of the PRS for each individual among the controls, and the mean absolute risk across all patients is presented stratified by ETS exposure (never, at home or at work, at home and at work). Additive tests for interactions between individual SNVs (0, 1, 2) and the PRS (80th vs 20th percentile) with ETS were computed using the additive.test function in R package CGEN (R Foundation for Statistical Computing).^26^ The function performs a likelihood ratio test for gene-environment interaction under an additive risk model, using a logistic regression, as well as tests under gene-environment independence assumption, using the retrospective likelihood.^27^ Significance testing was 2-sided, and *P* values <.05 were considered statistically significant; and all analyses were performed using R, version 4.2.2 statistical software.

## Results

Data were obtained on 1024 women with LUAD (mean [SD] age, 59.6 [11.4] years, 47.9% ever exposed to ETS at home, and 19.5% ever exposed to ETS at work) and 1024 controls (mean [SD] age, 58.9 [11.0] years, 37.0% ever exposed to ETS at home, and 14.3% ever exposed to ETS at work). We found that ETS exposure at home (odds ratio [OR], 1.49; 95% CI, 1.24-1.80) and at work (OR, 1.41; 95% CI, 1.11-1.81) were associated with an increased risk of LUAD. Furthermore, we found an exposure-response association between the PRS and LUAD risk (OR per SD increase of the PRS, 1.60; 95% CI, 1.46-1.75). The distributions of exposure to ETS and the PRS in GELAC are presented in eTable 3 and the eFigure in Supplement 1.

**Figure 1.**
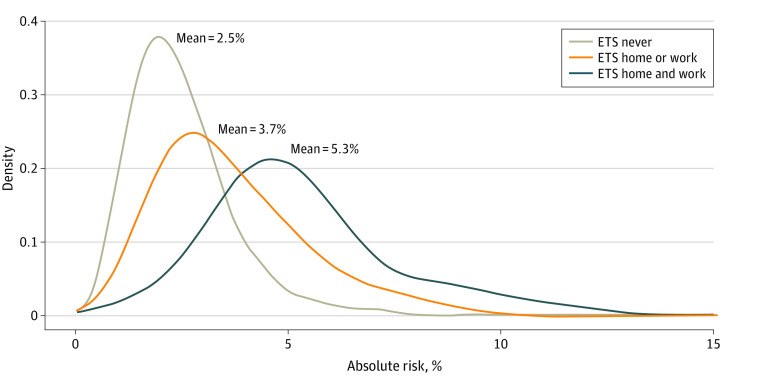
Density Plots for Estimates of Lifetime (Age Interval 40-80 Years) Absolute Risk of Lung Adenocarcinoma for Never-Smoking Female Controls in the Genetic Epidemiological Study of Lung Adenocarcinoma Study Based on Exposure to Environmental Tobacco Smoke (ETS) and Genetic Polygenic Risk Score (PRS)

We estimated the lifetime 40-year absolute risk of LUAD for never-smoking female controls in GELAC by integrating the PRS, ETS exposure, and lung cancer incidence in Taiwan, overall and by PRS decile categories. The estimated overall average lifetime 40-year absolute risk of LUAD was 2.5% (range, 0.6%-10.3%) among women never exposed to ETS. When integrating both ETS and PRS data, the estimated absolute risk of LUAD was 3.7% (range, 0.6%-14.5%) for women exposed to ETS either at home or work, and 5.3% (range, 1.2%-12.1%) for women exposed to ETS at both home and work (Figure 1). These findings suggest that there is a greater range in the absolute risk of LUAD and better ability to stratify risk when the PRS is included in the absolute risk calculations compared with estimates with ETS exposure alone or vice versa. Notably, women never exposed to ETS who have a high PRS may have a greater absolute lifetime risk of LUAD compared with women who are exposed to ETS with a low PRS. Similarly, the absolute risk of LUAD calculated with ETS and the PRS was nearly double that for women exposed to ETS who have high PRS compared with the average absolute risk calculated with ETS exposure alone.

**Figure 2.**
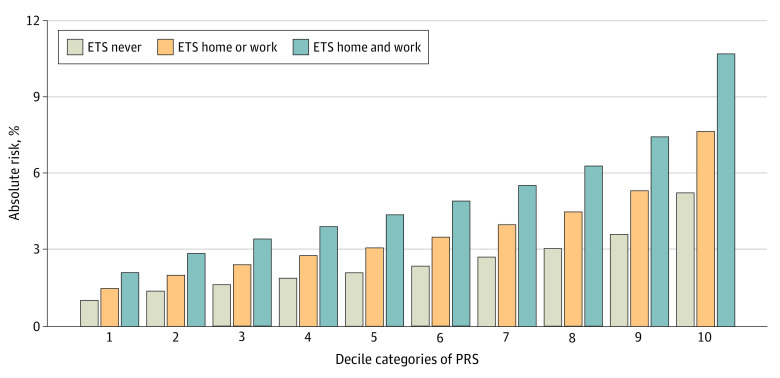
Bar Plots for Estimates of Lifetime (Age Interval 40-80 Years) Absolute Risk of Lung Adenocarcinoma for Never-Smoking Female Controls in the Genetic Epidemiological Study of Lung Adenocarcinoma Study Based on Exposure to Environmental Tobacco Smoke (ETS) and Genetic Polygenic Risk Score (PRS)

We observed that the difference in the average lifetime 40-year absolute risk of LUAD comparing women with high and low ETS exposure increases with higher PRS deciles (Table 1, Figure 2). We further identified these findings through an observed super-additive interaction between the PRS (80th vs 20th percentile), ETS (at home and at work vs never) and absolute risk of LUAD (*P* = 6.5 × 10^−4^ for interaction). Additionally, in analyses assessing gene-environment interaction between individual SNVs and ETS exposure, we observed a statistically significant super-additive interaction between ETS and rs2736100 (*P* = .01 for interaction) located on chromosome 5p15.33 near the *CLPTM1L* and *TERT* genes, at the 0.05 α threshold (Table 2). However, the association was no longer statistically significant after Bonferroni correction for multiple comparisons. A sensitivity analysis showed a statistically significant super-additive interaction between ETS and the PRS excluding rs2736100 with the risk of LUAD (*P* = .03 for interaction), suggesting the interaction is not driven by one locus.

**Table 1.** Average Estimates of Lifetime Absolute Risk of Lung Adenocarcinoma for Never-Smoking Female Controls in GELAC^a^

PRS decile	Average absolute risk (%)		
	ETS never	ETS home or work	ETS home and work
1	1.0	1.5	2.1
2	1.4	2.0	2.8
3	1.6	2.4	3.4
4	1.9	2.8	3.9
5	2.1	3.1	4.4
6	2.4	3.5	4.9
7	2.7	4.0	5.6
8	3.1	4.5	6.3
9	3.6	5.3	7.4
10	5.3	7.7	10.7

Abbreviations: ETS, environmental tobacco smoke; GELAC, Genetic Epidemiological Study of Lung Adenocarcinoma; PRS, polygenic risk score.

^a^ Age intervals from 40 to 80 years.

**Table 2.** Assessment of Additive Interaction Between Individual SNVs, Polygenic Risk Score, and Exposure to Environmental Tobacco Smoke With Lung Adenocarcinoma in 1024 Patients and 1024 Controls

SNV	CHR	BP	Genes	Eff/Ref	MAF (Ca/Co)	*P* value for interaction^a^
Polygenic risk score						6.5 × 10^−4^
rs2736100	5	1286516	*CLPTM1L*, *TERT*	C/A	0.50/0.38	.01
rs55768116	11	118108331	*AMICA1*	A/C	0.49/0.53	.05
rs9380190	6	30769565	*MHC*	C/T	0.43/0.42	.06
rs531557	6	53389995	*GCLC*	A/T	0.45/0.41	.06
rs11196089	10	114509290	*VTI1A*	C/T	0.34/0.29	.10
rs764014	15	56454223	*RFX7*	G/A	0.41/0.44	.27
rs9367106	6	41483390	*FOXP4*	C/G	0.33/0.33	.34
rs4268071	7	124373384	*GPR37*	T/G	0.30/0.28^b^	.35
rs6937083	6	117785308	*ROS1*/DCBLD1	T/A	0.46/0.52	.37
rs55779747	3	189354127	*TP63*	C/A	0.44/0.50	.48
rs17038564	2	65496058	*ACTR2*	G/A	0.22/0.20	.58
rs2293607	3	169482335	*LRRC34*	T/C	0.48/0.46	.59
rs71467682	15	49757466	*FGF7*, SE*CISBP2L*	G/A	0.28/0.29	.65
rs682888	2	25757709	*DTNB*	T/C	0.46/0.46	.73
rs59956089	17	65960854	*BPTF*	C/T	0.28/0.29^b^	.75
rs10901793	10	126324209	*FAM53B*, *METTL10*	A/G	0.32/0.30	.88
rs1373058	4	157894892	*PDGFC*	A/T	0.38/0.38^b^	.91
rs1200399	14	35293185	*BAZ1A*	T/C	0.46/0.49	.92
rs174559	11	61581656	*FADS1*	G/A	0.45/0.41	.96
rs7962469	12	52348259	*ACVR1B*	G/A	0.27/0.30^b^	.97
rs2760995	6	32574358	*MHC*	A/G	0.11/0.11	NA
rs72658409	9	22160087	*CDKN2B-AS1*	T/C	0.06/0.08	NA
rs137884934	3	138570011	*PIK3CB*	T/C	0.07/0.07	NA
rs116863980	19	725066	*PALM*	A/G	0.05/0.04	NA
rs117715768	4	44174404	*KCTD8*	T/C	0.09/0.08	NA

Abbreviations: BP, base pair; Ca, cases; CHR, chromosome; Co, controls; Eff, effect allele; MAF, minor allele frequency; NA, not applicable; Ref, reference allele; SNV, single nucleotide variant.

^a^ *P* values for additive interaction between each SNV (0, 1, 2), polygenic risk score (80th vs 20th percentile) and exposure to environmental tobacco smoke (never vs at home and work) were calculated using the likelihood ratio test using the additive.test function within the CGEN package in R. The function assumes independence between the genetic and environmental risk factors. All models were adjusted for age (continuous) and top 10 eigenvectors for population stratification. SNVs with missing *P* values for interaction (NA) had empty cells to assess for interaction.

^b^ Minor allele does not equal reference allele.

## Discussion

In this study, we observed the added benefit of integrating data on both exposure to ETS and genetic susceptibility to estimate the lifetime absolute risk of LUAD in never-smoking Asian women aged 40 years over a 40-year span. We estimated the overall average cumulative 40-year absolute risk of LUAD was 2.5% among women never exposed to ETS. When integrating both ETS and PRS data, the estimated average absolute risk of LUAD increased to 3.7% for women exposed to ETS either at home or work, and 5.3% for women exposed to ETS at both home and work. We also found that the estimated absolute risk of LUAD was higher among those with ETS exposure and high genetic susceptibility compared with those with ETS exposure and low genetic susceptibility. Specifically, we found that the difference in the absolute risk of LUAD between women exposed and unexposed to ETS is greater among those with high PRS vs low PRS. This association was confirmed through a significant super-additive interaction between ETS exposure and the PRS, as well as ETS exposure and a key SNV on the *TERT* gene. To our knowledge, this is one of the first studies to estimate the absolute risk of LUAD attributed to ETS exposure stratified by genetic susceptibility.

Assessing absolute risk of lung cancer and understanding the burden of its risk factors is crucial for developing health interventions. Given this, developing absolute risk models can help identify individuals who are at high risk for lung cancer who can potentially benefit from targeted primary and secondary disease prevention strategies, such as prioritizing reduction of exposure to risk factors and lung cancer screening.^16^ Our work supports that incorporating a PRS that captures genetic susceptibility in absolute risk stratification can identify subgroups of never-smokers with a high risk of LUAD that may not have been identified in models with ETS exposure alone. Furthermore, the differences in the absolute risk of LUAD due to ETS at low and high percentiles of the PRS may indicate that increased exposure to ETS may confer higher absolute risk of LUAD among women who are more genetically susceptible and whom may most benefit from targeted intervention efforts.

We found a suggestive super-additive interaction between exposure to ETS and an SNV on the telomerase reverse transcriptase (*TERT*) gene (rs2736100), suggesting the association between ETS and LUAD may be modified by genetic susceptibility. The *TERT* gene is 1 of 2 major telomerase genes that maintain telomere DNA length, chromosomal stability, and cellular immortality. Telomeres are functional structures at the end of chromosomes designed to maintain the stability and integrity of the genome, and whose dysfunction may lead to chromosomal instability and increased susceptibility to cancer.^28,29^ A previous study found that longer telomere length measured phenotypically in white blood cells was associated with an increased risk of lung cancer in 3 separate cohorts among smokers and never-smokers.^30^ We also observed that the C allele in rs2736100 is associated with longer telomere length measured phenotypically in a population of never-smoking women of East Asian ancestry,^31^ as well as with the risk of lung cancer.^18,19,32,33,34,35,36,37^ The super-additive interaction in our study suggests that the effect of ETS on LUAD risk in never-smokers is greater with the presence of the rs2736100 C allele. Additional work is needed to identify the potential mechanisms of the modification by rs2736100 in the association between ETS and the risk of LUAD.

### Strengths and Limitations

Our study has some strengths. First, we built a PRS using SNVs from what is, to our knowledge, the most recent and largest 2-stage multiancestry GWAS of lung cancer with 21 658 cases of histologically confirmed LUAD in East Asian populations conducted to date. Second, we conducted our analyses in one of the largest studies of never-smoking women with histologically confirmed LUAD cases, for which both questionnaire-based and genetic data were collected.

This study also has limitations. Given that the samples from GELAC were part of the GWAS that identified the SNVs used in the PRS, the main effect of the PRS may be slightly overestimated due to winner’s curse. Additionally, we were not able to incorporate age-specific mortality rates, which may have resulted in a slight overestimation of the absolute risk. However, we expect the added risk stratification of including genetic susceptibility in the absolute risk, as well as the difference in the effect of the PRS stratified by ETS exposure, would still be observed. Furthermore, as we were only able to calculate projected risk based on estimated ORs, an independent cohort is needed to calibrate the risk model and calculate empirical risk.

## Conclusions

This case-control study presents preliminary findings for the stratification of absolute risk of LUAD in never-smoking women in Asia using integrated data from ETS exposure and genetic susceptibility. Future studies on the genetic architecture of lung cancer in never-smoking individuals in East Asia are needed to further characterize the underlying genetic basis of the disease. Further studies are warranted to expand on our findings by incorporating additional risk factors to build and validate risk prediction models in a representative population to better inform the development of lung cancer screening programs for never-smokers.

## Notes

Supplement 1. **eTable 1.** Independent Single Nucleotide Polymorphisms Identified in a Previously Conducted Genome-Wide Association Study and Included in the Polygenic Risk Score **eTable 2.** Average Lung Cancer Incidence Among Never-Smokers in Taiwan from 2002-2010 **eTable 3.** Characteristics of the Genetic Epidemiological Study of Lung Adenocarcinoma (GELAC) in Taiwan **eFigure.** Distribution of the Polygenic Risk Score By Case-Control Status in the Genetic Epidemiological Study of Lung Adenocarcinoma (GELAC) in Taiwan

Supplement 2.
Data Sharing Statement
