# Supplementary material for: Polygenic Risk Score, Environmental Tobacco Smoke, and Risk of Lung Adenocarcinoma in Never-Smoking Women in Taiwan
Source: JAMA Netw Open. 2023 Nov 13;6(11):e2339254. doi: 10.1001/jamanetworkopen.2023.39254 (PMC10644212; doi:10.1001/jamanetworkopen.2023.39254)
Supplement: Supplement 1. — eTable 1. Independent Single Nucleotide Polymorphisms Identified in a Previously Conducted Genome-Wide Association Study and Included in the Polygenic Risk Score eTable 2. Average Lung Cancer Incidence Among Never-Smokers in Taiwan from 2002-2010 eTable 3. Characteristics of the Genetic Epidemiological Study of Lung Adenocarcinoma (GELAC) in Taiwan eFigure. Distribution of the Polygenic Risk Score By Case-Control Status in the Genetic Epidemiological Study of Lung Adenocarcinoma (GELAC) in Taiwan [file jamanetwopen-e2339254-s001.pdf]

Supplementary Online Content

Blechter B, Chien LH, Chen TY, et al. Polygenic risk score, environmental tobacco smoke, and risk of lung adenocarcinoma in never-smoking women in Taiwan. *JAMA Netw Open*. 2023;6(10):e2339254. doi:10.1001/jamanetworkopen.2023.39254

eTable 1. Independent Single Nucleotide Polymorphisms Identified in a Previously Conducted Genome-Wide Association Study and Included in the Polygenic Risk Score

eTable 2. Average Lung Cancer Incidence Among Never-Smokers in Taiwan from 2002-2010

eTable 3. Characteristics of the Genetic Epidemiological Study of Lung Adenocarcinoma (GELAC) in Taiwan

eFigure. Distribution of the Polygenic Risk Score By Case-Control Status in the Genetic Epidemiological Study of Lung Adenocarcinoma (GELAC) in Taiwan

This supplementary material has been provided by the authors to give readers additional information about their work.

© 2023 Blechter B et al. *JAMA Network Open.*

eTable 1. Independent single nucleotide polymorphisms identified in a previously conducted genome-wide association study and included in the polygenic risk score

SNP	CHR	BP	Genes	Eff/Ref	MAF	ORt[a]
rs10901793	10	126324209	*FAM53B, METTL10*	A/G	0.30	1.21
rs11196089	10	114509290	*VTI1A*	C/T	0.30	1.30
rs116863980	19	725066	*PALM*	A/G	0.06	1.16
rs117715768	4	44174404	*KCTD8*	T/C	0.06	1.17
rs1200399	14	35293185	*BAZ1A*	T/C	0.50	0.91
rs1373058	4	157894892	*PDGFC*	A/T	0.43	1.08
rs137884934	3	138570011	*PIK3CB*	T/C	0.09	0.82
rs17038564	2	65496058	*ACTR2*	G/A	0.20	1.08
rs174559	11	61581656	*FADS1*	G/A	0.39	0.92
rs2293607	3	169482335	*LRRC34*	T/C	0.46	0.88
rs2736100	5	1286516	*CLPTM1L, TERT*	C/A	0.39	0.74
rs2760995	6	32574358	*MHC*	A/G	0.13	1.18
rs4268071	7	124373384	*GPR37*	T/G	0.04	1.38
rs531557	6	53389995	*GCLC*	A/T	0.40	0.92
rs55768116	11	118108331	*AMICA1*	A/C	0.42	0.88
rs55779747	3	189354127	*TP63*	C/A	0.47	0.81
rs59956089	17	65960854	*BPTF*	C/T	0.30	1.18
rs682888	2	25757709	*DTNB*	T/C	0.47	0.90
rs6937083	6	117785308	*ROS1/DCBLD1*	T/A	0.48	0.82
rs71467682	15	49757466	*FGF7, SECISBP2L*	G/A	0.31	0.91
rs72658409	9	22160087	*CDKN2B-AS1*	T/C	0.09	0.74
rs764014	15	56454223	*RFX7*	G/A	0.47	0.91
rs7962469	12	52348259	*ACVR1B*	G/A	0.34	1.16
rs9367106	6	41483390	*FOXP4*	C/G	0.34	1.20
rs9380190	6	30769565	*MHC*	C/T	0.30	0.89

Abbreviations: SNP, single nucleotide polymorphism; CHR, chromosome, BP, base pair; Eff, effect allele; Ref, reference allele; MAF, minor allele frequency; OR, odds ratio

[a]Log(OR) values used to weigh corresponding SNPs in the polygenic risk score obtained from a recently conducted genome-wide association study by Shi et al. 2023

© 2023 Blechter B et al. *JAMA Network Open.*

eTable 2. Average lung cancer incidence among never-smokers in Taiwan from 2002-2010

Age	Lung cancer incidence (per 100,000)*
40	7.34889
41	9.83000
42	10.63222
43	12.50111
44	15.72778
45	15.07889
46	17.12556
47	19.88778
48	23.00778
49	25.45556
50	25.67444
51	30.61778
52	33.09111
53	38.30556
54	38.32778
55	44.40444
56	49.81111
57	52.33444
58	58.43000
59	64.27889
60	68.65000
61	75.04556
62	72.66889
63	74.81778
64	80.03667
65	94.70444
66	97.19556
67	106.17000
68	109.42444
69	126.81111
70	127.31111
71	137.22222
72	146.82667
73	165.51333
74	162.35556
75	177.05333
76	185.34667

© 2023 Blechter B et al. *JAMA Network Open.*

77	177.70222
78	184.79556
79	213.70222
80	216.52556

*Data on lung cancer incidence rates in Taiwan were obtained from Chien et al. 2020

© 2023 Blechter B et al. *JAMA Network Open.*

eTable 3. Characteristics of the Genetic Epidemiological Study of Lung Adenocarcinoma (GELAC) in Taiwan

	Cases (N=1,024)	Controls (N=1,024)
Age, Mean (SD)	59.6 (11.4)	58.9 (11.0)
ETS exposure at home, n (%)		
Never	534 (52.1%)	645 (63.0%)
Ever	490 (47.9%)	379 (37.0%)
ETS exposure at work, n (%)		
Never	824 (80.5%)	878 (85.7%)
Ever	200 (19.5%)	146 (14.3%)

Abbreviations: SD, standard deviation; ETS, environmental tobacco smoke

© 2023 Blechter B et al. *JAMA Network Open.*

eFigure. Distribution of the polygenic risk score by case-control status in the Genetic Epidemiological Study of Lung Adenocarcinoma (GELAC) in Taiwan.

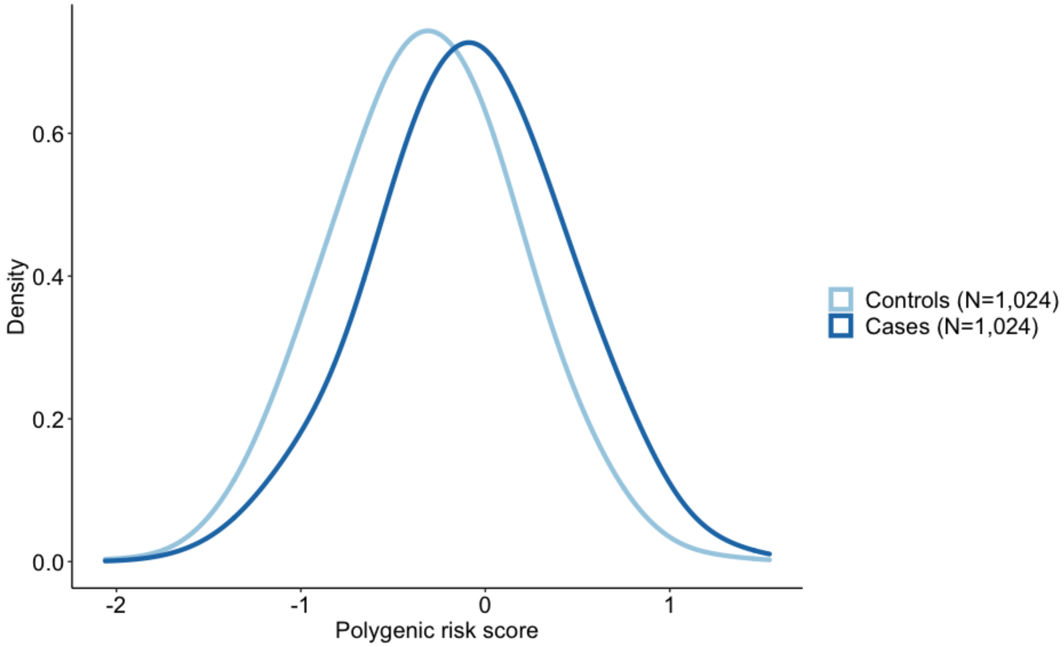

© 2023 Blechter B et al. *JAMA Network Open.*
