# Supplementary material for: Polygenic Risk Score, Environmental Tobacco Smoke, and Risk of Lung Adenocarcinoma in Never-Smoking Women in Taiwan
Source: JAMA Netw Open. 2023 Nov 13;6(11):e2339254. doi: 10.1001/jamanetworkopen.2023.39254 (PMC10644212; doi:10.1001/jamanetworkopen.2023.39254)
Supplement: Supplement 2. — Data Sharing Statement [file jamanetwopen-e2339254-s002.pdf]

Data Sharing Statement

Blechter. Polygenic Risk Score, Environmental Tobacco Smoke, and Risk of Lung Adenocarcinoma in Never-Smoking Women in Taiwan. *JAMA Netw Open*. Published November 13, 2023. doi:10.1001/jamanetworkopen.2023.39254

Data
Data available: No

Additional Information
Explanation for why data not available: The genome-wide association study data are deposited at dbGAP (https://www.ncbi.nlm.nih.gov/gap, study accession: phs000716.v1.p1). Additional data can be made available upon request.
